# The true costs of cesarean delivery for patients in rural Rwanda: Accounting for post-discharge expenses in estimated health expenditures

**DOI:** 10.1186/s12939-022-01664-x

**Published:** 2022-05-08

**Authors:** Anne Niyigena, Barnabas Alayande, Laban Bikorimana, Elizabeth Miranda, Niclas Rudolfson, Deogratias Ndagijimana, Fredrick Kateera, Robert Riviello, Bethany Hedt-Gauthier

**Affiliations:** 1 Partners In Health, Kigali, Rwanda; 2 University of Global Health Equity, Butaro, Rwanda; 3 Program in Global Surgery and Social Change, Harvard Medical School, Boston, MA USA; 4 Division of Vascular Surgery, University of Southern California, Los Angeles, CA USA; 5 WHO Collaborating Centre for Surgery and Public Health, Lund University, Lund, Sweden; 6 Department of Global Health and Social Medicine, Harvard Medical School, Boston, MA USA; 7 Center for Surgery and Public Health, Brigham and Women’s Hospital, Boston, MA USA

**Keywords:** Cost of post-operative care, Healthcare cost, Follow-up care, Catastrophic health expenditure, Cesarean, c-section, Rural, Health insurance, Poverty, Healthcare access

## Abstract

**Introduction:**

While it is recognized that there are costs associated with postoperative patient follow-up, risk assessments of catastrophic health expenditures (CHEs) due to surgery in sub-Saharan Africa rarely include expenses after discharge. We describe patient-level costs for cesarean section (c-section) and follow-up care up to postoperative day (POD) 30 and evaluate the contribution of follow-up to CHEs in rural Rwanda.

**Methods:**

We interviewed women who delivered via c-section at Kirehe District Hospital between September 2019 and February 2020. Expenditure details were captured on an adapted surgical indicator financial survey tool and extracted from the hospital billing system. CHE was defined as health expenditure of ≥ 10% of annual household expenditure. We report the cost of c-section up to 30 days after discharge, the rate of CHE among c-section patients stratified by in-hospital costs and post-discharge follow-up costs, and the main contributors to c-section follow-up costs. We performed a multivariate logistic regression using a backward stepwise process to determine independent predictors of CHE at POD30 at α ≤ 0.05.

**Results:**

Of the 479 participants in this study, 90% were classified as impoverished before surgery and an additional 6.4% were impoverished by the c-section. The median out-of-pocket costs up to POD30 was US$122.16 (IQR: $102.94, $148.11); 63% of these expenditures were attributed to post-discharge expenses or lost opportunity costs (US$77.50; IQR: $67.70, $95.60). To afford c-section care, 64.4% borrowed money and 18.4% sold possessions. The CHE rate was 27% when only considering direct and indirect costs up to the time of discharge and 77% when including the reported expenses up to POD30. Transportation and lost household wages were the largest contributors to post-discharge costs. Further, CHE at POD30 was independently predicted by membership in community-based health insurance (aOR = 3.40, 95% CI: 1.21,9.60), being a farmer (aOR = 2.25, 95% CI:1.00,3.03), primary school education (aOR = 2.35, 95% CI:1.91,4.66), and small household sizes had 0.22 lower odds of experiencing CHE compared to large households (aOR = 0.78, 95% CI:0.66,0.91).

**Conclusion:**

Costs associated with surgical follow-up are often neglected in financial risk calculations but contribute significantly to the risk of CHE in rural Rwanda. Insurance coverage for direct medical costs is insufficient to protect against CHE. Innovative follow-up solutions to reduce costs of patient transport and compensate for household lost wages need to be considered.

## Introduction

Access to emergency obstetric care, including cesarean sections (c-sections), is an essential part of a functional health system [1]. However, inequalities in c-section access have been reported across and within countries [2, 3]. Financial barriers are the most cited driver of c-section inaccessibility [4], with poor access most affecting the economically deprived [5–7]. Limited access to c-sections is associated with higher risks of poor outcomes for mothers and their babies [4, 8].

C-sections are considered cost-effective interventions, costing US$251 to US$3,462 per disability adjusted life year saved [9]. However, women who deliver via c-section are at risk of financial hardship. Studies in sub-Saharan Africa have estimated the direct costs of c-section to be $144-$426 [10–12], a considerable amount compared to the average regional gross domestic product (GDP) per capita of $4,195 in 2019 [13]. Even when direct costs are heavily subsidized, indirect costs put a woman and her family in financial risks [14]. Studies have shown that surgery in general [9, 15, 16], and c-sections specifically [17, 18], can be financially catastrophic for a patient’s family. However, these studies fail to include the extended costs for surgery, and potentially underestimating the true risk of catastrophic health expenditures (CHEs) due to surgery.

In Rwanda, the location of this study, 28% of patients undergoing peritonitis surgery suffered CHEs as a result of the surgery [19]. While these studies include direct and indirect medical costs up to the time of discharge, we are unaware of any study of surgery in Africa that considers costs associated with postoperative follow-up after discharge. In this paper, we describe the financial costs of c-section care for Rwandan women delivering via c-section at a rural district hospital, including direct and indirect costs of all care received up to postoperative day (POD 30), and estimate the full risk of CHE for these women.

## Methods

### Study setting

This study was nested in a prospective cohort study conducted at Kirehe District Hospital, which aimed to evaluate the feasibility and acceptability of a telemedicine intervention for the diagnosis of post-hospital discharge surgical site infections by community health workers. Kirehe District Hospital is located in the Eastern Province of Rwanda and is managed by Rwanda’s Ministry of Health with technical support from Partners In Health/Inshuti Mu Buzima (PIH/IMB), a Boston-based non-governmental organization that provides technical support to the Ministry of Health.

In Rwanda, c-sections are typically performed at district hospitals by general practitioners (GPs) [20], and at Kirehe District Hospital, c-section is the most commonly performed surgery. After delivery, women are monitored in a post-c-section ward and usually discharged on POD 3. In Rwanda, there are no standardized guidelines for c-section follow-up; however, at Kirehe District Hospital, c-section patients are asked to visit the local peripheral health centers three days post-discharge for wound inspection and dressing change and to continue follow-up until deemed unnecessary by the health center nurse.

Approximately 83% of the Rwandan population has health insurance and 96.1% of insured rural residents are enrolled in the community-based health insurance (CBHI) program [21]. Rwanda's CBHI is based on a 4-tier wealth system called *Ubudehe*, with the bottom tier including the poorest and the upper tier including the wealthiest Rwandans. For those in *Ubudehe* 1, CBHI premium is fully subsidized by the government; individuals in *Ubudehe* 2 and 3 pay CHBI premiums of approximately US$3 per person each year and those in *Ubudehe* 4 pay a premium of US$7 per person per year[22]. Individuals in *Ubudehe* 1 pay no copayment at point of care while those in *Ubudehe 2–4* incur a 10% copayment for direct medical services.

### Sample selection

We used a convenient sample of women who delivered via c-section at Kirehe District Hospital between September 23rd, 2019 and February 22nd, 2020, who were eligible for and enrolled in a prospective cohort study aiming to evaluate the feasibility and acceptability of a telemedicine intervention for the diagnosis of post-hospital discharge surgical site infections by community health workers.

### Data collection

#### Enrollment data collection

Women were enrolled after c-section delivery and prior to discharge. All participants provided informed consent prior to data collection. Data collectors administered sociodemographic and clinical characteristics questionnaires before patients were discharged from the hospital; data were directly entered into REDCap data management software [23]. Patients also responded to a financial survey, described below. Data on healthcare expenditures was extracted from OpenMRS, an online database tracking details on patients’ medical care and expenses.

#### Follow-up data collection

At enrollment, respondents provided cell phone numbers (their own, a relative’s or a neighbor’s) on which they could be contacted. On POD 30 (± 1 day), data collectors administered a phone-based follow-up interview to assess post-discharge follow-up activities. The costs of post-discharge c-section follow-up were assessed in terms of expenses for medical care, expenses for transport, and lost wages due to seeking follow up care at the health centers. Study participants that could not be reached by the phone number they provided at discharge were contacted in person by a local community health worker and a telephone survey was administered on the community health worker’s telephone. Three attempts on three different days were made in an effort to maximize the response rate; individuals not contacted after three attempts were considered lost-to-follow-up. The POD30 response rate was 84%.

#### Financial Survey

Our financial survey was adapted from a standardized financial questionnaire developed and validated through the National Surgical Obstetric Anesthesia Planning by the Program in Global Surgery and Social Change [24]. We added the following variables to the core questionnaire: estimates of monthly household income, self-reported routine monthly household expenditure, whether the patient had to borrow money or sell possessions to pay for the current hospitalization, and household monthly consumption as a sum of expenditures for food and drink, transportation, livestock, housing, transportation fees, school fees, and healthcare in the past months. Non-monetary income such as agricultural harvest was converted into Rwandan Franc (RWF) using the price of local goods at the time of data collection. Lost wages were estimated using daily wages for the occupation of the patients and caregiver at the time, and reported in RWF.

### Definition of key terms

We stratified expenses into two main categories: in-hospital costs and post-discharge follow-up costs. The in-hospital costs include expenditures from when a woman left her home to seek care for delivery until the time of discharge. In-hospital costs were further grouped into direct medical, direct non-medical, and indirect costs. Direct medical costs included payments for medical supplies, medications, laboratory exams, surgical procedure, imaging, consultation, and hospital bed. Direct non-medical costs included expenses of a caregiver during hospitalization, food and transport from home to hospital. Indirect costs included household lost wages due to hospitalization. Post-discharge follow-up costs included direct medical cost paid at the health center in addition to indirect follow-up costs. The direct non-medical follow-up costs included transport from hospital to home for the patient and caregiver, transportation to the health center for patient and caregiver; while indirect follow-up costs included lost wages due to delayed return to work and lost wages of both the patient seeking follow-up care and that of the accompanying caregiver. We chose to include the cost of transport from the hospital to home after the c-section in the follow-up care costs as these expenses are generally not factored in CHE studies and allows for direct comparability of our results.

Poverty was defined using the World Bank definitions, defined as a daily expenditure below $1.90 per person per day [25]. Catastrophic health expenditure (CHE) has been variously defined as out-of-pocket healthcare expenses that exceeds 10% of total annual household expenditure or income [26, 27], or as spending greater than 40% of the annual household income, excluding subsistence needs, on health care [28]. For this study, we defined CHE as healthcare spending of greater than 10% of annual household consumption to align with the definition of the United Nations’ Sustainable Development Goals 3.8.2 [29]. The annual household consumption was defined as a sum of annual expenditures on food and drink, transportation, livestock, housing, transportation fees, school fees, healthcare and other expenses.

### Statistical analysis

We restricted our analysis to patients who responded to the financial questionnaires at both time points. We also restricted our analyses to individuals who sought follow-up care at the health center at least once during the first 30 postoperative days so we could estimate the costs associated to follow-up care. All tradeable financial expenses, such as in-hospital expenses and transport fees, were converted into US dollars (US$) using the nominal exchange rate at study start date (October, 2019), and US$1 equated RWF916.17 [30]. All non-tradeable expenses, including income and lost wages were converted to US$ using the 2019 Rwanda purchasing power parity (PPP) conversion factor for personal consumption of 317.18 [31].

We describe our sample, focusing on the demographic characteristics, household characteristics, and clinical features most relevant to understanding the study population and resources and complexity of c-section recovery, using frequency and percentages for categorical variables, mean and standard deviation (SD) for normally distributed continuous variables, and median and interquartile range (IQR) for continuous variables with non-normal distributions. We summarize the financial cost of c-section care stratified by Rwanda’s four-tier wealth classification by in-hospital and follow-up care components, using median and interquartile range. We summarize each of the main cost contributors as a percentage of the overall costs. We also calculated incidence of CHE for all expense categories and reported the frequencies and percents. We conducted Chi-square tests for categorical predictor variables and Wilcoxon rank sum test for continuous predictors to assess the association between CHE and patients’ characteristics. We then performed a Wald test for multivariate logistic regression to assess independent predictors of CHE at POD30. The bivariate analysis included pre-cesarean section characteristics such as: age, education, type of insurance, occupation, Ubudehe category parity, number of antenatal care visits, number of prior c-sections, mode of transport to health facility, travel time from home to health center, travel time from health center to the hospital and household size. All variables significant at the α = 0.20 significance level were considered for the full model; we then reduced the full model using a backward stepwise process and removed non-statistically significant variables one at the time until the final model remained with variables significant at α = 0.05 significance level. Our final regression model is specified as:

$$\mathrm{log}\frac{p}{1-p}={\beta }_{0}+{\sum }_{i=1}^{m}{\beta }_{i}{x}_{i}$$

where ${x}_{i}$ are the covariates retained in the model corresponding to the coefficients, ${\beta }_{i}$, that remain significant at the α = 0.05 significance level after the backward stepwise process. CHE at POD30 as a dichotomous variable, was the dependent variable in the multivariate regression analysis and level of education, main occupation, health insurance types and household size were the covariates that remained in the final reduced model.

We determined the daily expenditure per person in the household as a sum of individual expenses of the household divided by the household size, and report the proportion of participants who lived below the poverty line, before c-section delivery. We then calculated the total expenditure remaining after paying c-section cost, and estimated the proportion of women whose spending is below poverty line. The percentage of people who were pushed into extreme poverty by c-section delivery reflects people whose annual total expenditure were above poverty line at baseline, but who fell below poverty after paying for the costs of c-section care up through POD30.

## Results

In total, 479 patients were included in this study, of whom 68.7% were aged less than 30 years, and the majority (94.8%) were insured by CBHI (Table 1). All c-sections were performed at KDH by general practitioners. Approximately 10% of patients belonged to the lowest *Ubudehe* category, 84.7% were farmers and the median annual household income was US$532.8 (IQR: $232.8, $859.1). The median travel time from home to health center was 30 min (IQR: 15, 60 min) and from health center to hospital was 40 min (IQR: 5, 60 min). For the 433 (81%) patients who first sought care at the health center prior to going to Kirehe District Hospital, 61.6% were transported to the hospital in ambulance and 26.9% walked to the hospital.

**Table 1:** Characteristics of c-section patients at Kirehe District Hospital (*N* = 479)

	**Frequency**	**Percent**
	***N* = 479**	**(%)**
**SOCIODEMOGRAPHIC CHARACTERISTICS**		
**Age**		
< 18	17	3.6
18–30	312	65.1
31–40	131	27.3
> 40	19	4.0
**Level of education**		
Less than 6 years of primary school	48	10.0
Completed 6 years of primary school	314	65.6
Secondary school or more ^¥^	117	24.4
**Occupation**		
Farmer	406	84.7
Employed	54	11.3
Unemployed	19	4.0
Ubudehe* categories (*N* = 477)		
*Ubudehe 1*	44	9.2
*Ubudehe 2*	258	54.1
*Ubudehe 3 & 4*	175	36.7
**Insurance Type**		
Community Based Health Insurance or *Mutuelle*	454	94.8
Private	25	5.2
**CLINICAL CHARACTERISTICS**		
**Parity**		
Primiparous (1)	167	34.9
Multiparous (2–5)	277	57.8
Grand-multiparous (> 5)	35	7.3
**Antenatal Care Visits (*N* = 478)**		
1 to 4	473	98.9
Greater than 4	5	1.1
**Patients with postoperative complications**¶	10	2.1
**Number of post discharge follow up visits** Median (IQR)	2	(1–3)
**Number of prior c-section**		
None	325	68.4
1	111	23.4
2 or 3	39	8.2
**ACCESS TO HOSPITAL AND HEALTH CENTER**		
**Mode of transport (health center to hospital)**		
Ambulance	295	61.6
Walked	129	26.9
Public or private transport	17	3.1
**Travel times**		
Home to health center (min) Median (IQR)(*N* = 435)	30	(15,60)
Health center to hospital (min) Median (IQR)(*N* = 434)	40	(5,60)
**HOUSEHOLD FINANCIAL CHARACTERISTICS**		
**Household size**, Median (IQR)	4	(3,6)
**Self-reported annual income**, (USD), Median (IQR)	1510.4	(1028.0,2435.5)
**Self-reported annual household expenditure,** (USD)§, Median (IQR) *N* = 476	504.8	(331.8,751.8)
**Daily expenditure/person,** (USD)§ median (IQR)	0.3	(0.2–0.5)
**Living below international poverty line**ǂ	431	90.0
**Borrowed money to afford c-section expenses** *N* = 478	308	64.4
**Sold possessions to afford c-section expenses** (*N* = 474)	87	18.4

¥ Secondary school or more: include those who enrolled in high school but didn’t complete it, those who completed of high school, and those who have some years of education in a bachelor or masters degrees

^*^4-tier wealth system the bottom tier represents the least privileged and the upper tier is for the financially better off

^¶^ Post-operative complications include surgical site infections, post-partum hemorrhage, wound dehiscence and other wound complication

^§^Converted to USD using nominal exchange rate at study start date (October, 2019) 1 USD = 916.17 RWF

ǂInternational extreme poverty line = 1.9USD

The median household size was 4 people (IQR: 3, 6) (Table 1). The annual household expenditure was US$504.8 (IQR: $331.8, $751.8), translating to a daily expenditure of US$0.3 (IQR: $0.2, $0.5) per person. An overwhelming majority of women (90%) were from households living below the international extreme poverty line. Over half of participants (64%) borrowed money and 18.4% sold possessions to cover c-section related costs.

Table 2 summarizes the out-of-pocket costs of c-section by expense categories.

*Up to the time of discharge*, the median direct medical costs of cesarean section was US$8.8 (IQR: $8.0, $9.7) and indirect costs was USD$15.0 (IQR: $10.1, $21.7). The median total costs up to the time of hospital discharge was US$40.0 (IQR: $30.4, $55.7). These costs ranged from US$23.7 (IQR: $15.3, $38.3) for patients in *Ubudehe 1*, US$37.9 (IQR: $29.9, $54.3) for patients in *Ubudehe 2*, and US$46.0 (IQR: $35.9, $60.5) for patients in *Ubudehe 3 and 4*.

*For post-discharge costs*, the median number of follow-up visits at the health center was 2 (IQR: 1, 3) and the cost of medical bills was US$0.5 (IQR: $0.3, $0.7). The median post-discharge costs of c-section was US$71.4 (IQR: $60.7, $81.8) for patients in *Ubudehe 1*, US$77.5 (IQR: $67.7, $95.6) for patients in *Ubudehe 2*, US$79.7 (IQR: $71.1, $97.8) for patients in *Ubudehe 3 and 4.*

The total cost of c-section up to POD 30 was US$100.5 (IQR: $87.2, $118.4) for *Ubudehe 1*, US$119.4 (IQR: $102.6, $149.2) for *Ubudehe 2*, and US$134.1 (IQR: $113, $161.3) for *Ubudehe 3 and 4*. The cost of post-discharge expenses contributed 64.3% of these costs.

On average, 55.3% of the overall cost was taken up for post discharge lost wages, 15.8% for post discharge follow up at the health center, 13.3% for caregiving and 11.0% for transportation.

**Fig. 1:**
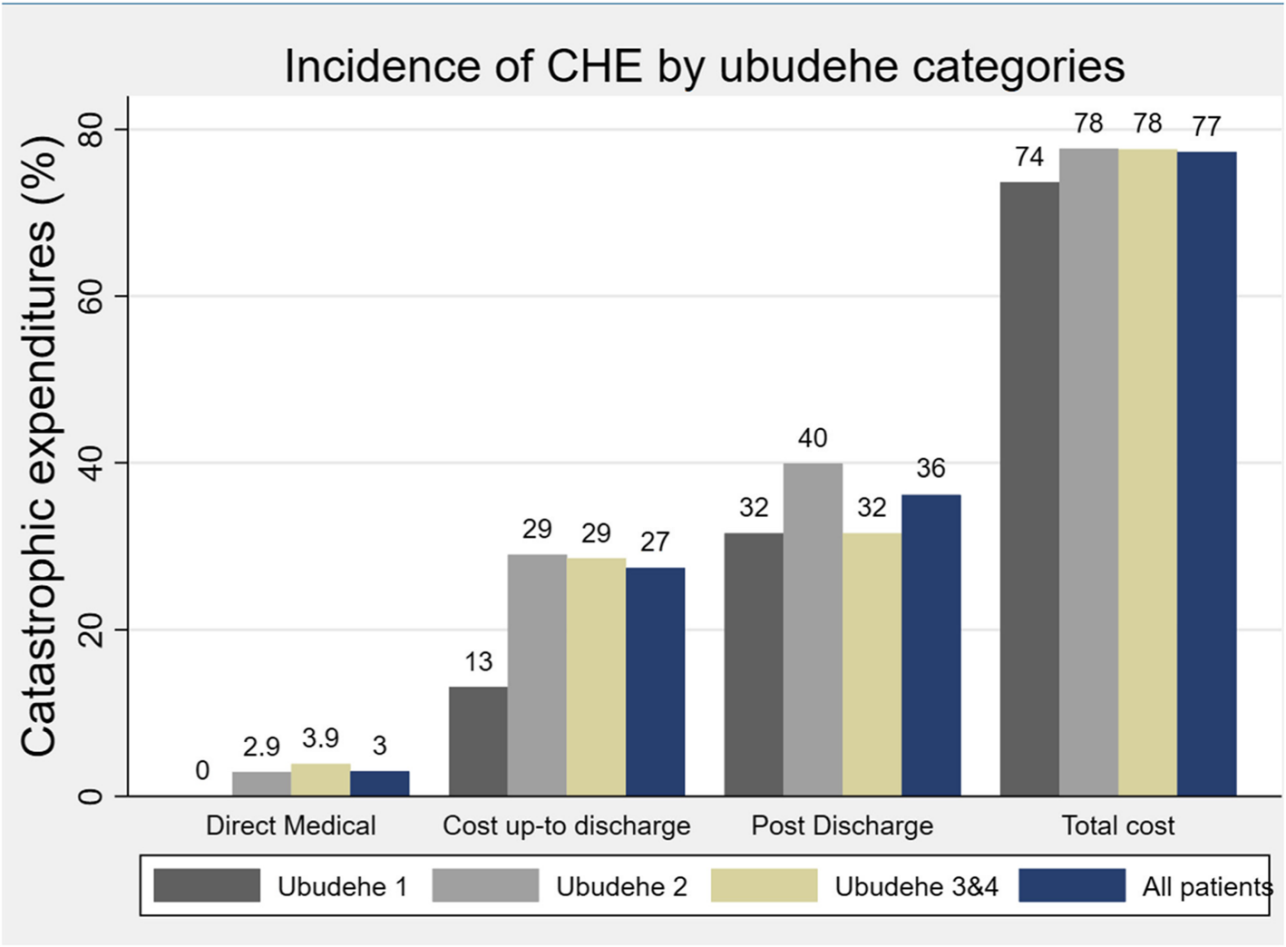
Incidence of catastrophic health expenditure by *Ubudehe* categories

When including only the costs up to the time of discharge, the cost of c-section was catastrophic for 3.0% of patients if only accounting for direct medical expenses and 27.0% when non-medical expenses were included **(**Fig. 1). Patients in *Ubudehe 1* had the lowest rate of CHE up to discharge (13.0%) compared to the rate of 29.0% for patients in *Ubudehe 2* and *Ubudehe 3 and 4.* When considering all costs through POD 30, the overall cost of c-section was catastrophic for 77.0% of patients. An additional 6.4% of patients were pushed into extreme poverty by the cost of the c-section.

From the bivariate analysis, factors associated with CHE that were identified as candidates for the multivariate logistic regression were: level of education (*p* < 0.01), occupation (*p* < 0.01), Ubudehe categories (*p* = 0.16), type of insurance (*p* < 0.01), parity (*p* = 0.17), mode of transport from health center to hospital (*p* = 0.03), travel time from home to health center (*p* = 0.01), and household size (*p* = 0.07). In a final reduced multivariate logistic regression model (Table3), women who completed primary school had 2.35 times higher odds to report CHE at POD30 than those who had secondary school enrollment or more (aOR = 2.35, 95%CI: 1.19, 4.66); farmers had 2.25 times higher odds to report CHE than employed women (aOR = 2.25, 95%CI: 1.00, 3.03); membership into community based health insurance was associated with 3.40 times higher odds of CHE (aOR = 3.40, 95%CI: 1.21,9.60); and small household sizes was associated with 22% lower odds of CHE compared to large household sizes (aOR = 0.78, 95%CI: 0.66,0.91).

**Table 2:**
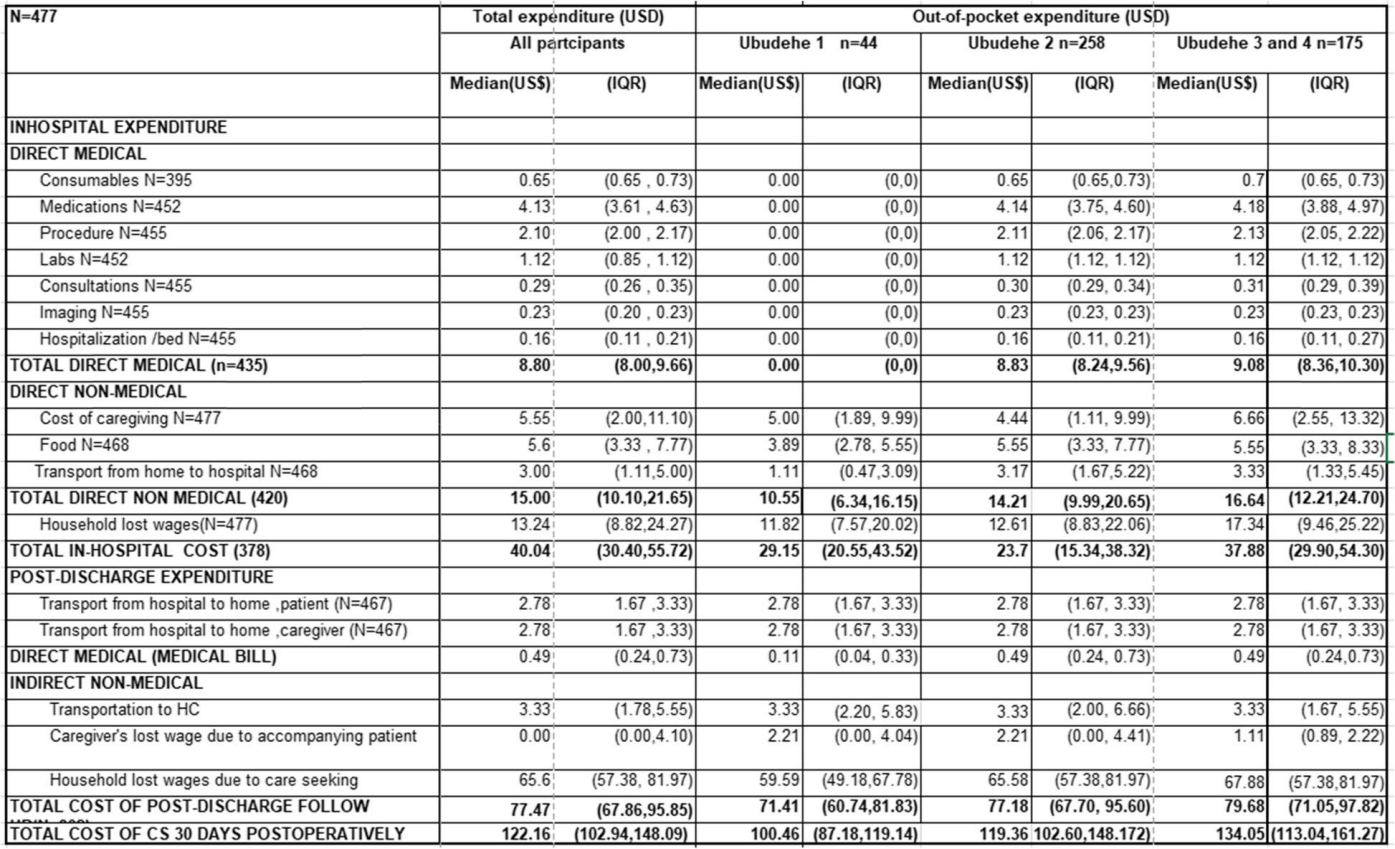
Summary of total and out-of-pocked expenditure for c-section hospitalization and follow up by *Ubudehe* categories (in US$) (N=477)

* Expenses are converted to USD using nominal exchange rate at study start date (October, 2019) 1 USD = 916.17 RWF

**Table 3:** Multivariate analysis of pre- cesarean section characteristics independently associated with CHE at POD30 (*N* = 428)

	**Full Model**			**Reduced Model**		
	**OR**	**95% CI**	***p*** **-value**	**OR**	**95% CI**	***p*** **-value**
**Level of education**						
Less than completed primary school	2.46	(0.61, 9.31)	0.18	3.09	(0.92,10.35)	0.06
Completed primary school	1.88	(0.78, 4.50)	0.03	2.35	(1.19, 4.66)	0.01
Secondary school or more	ref			ref		
**Occupation**						
Farmer	2.13	(0.82, 5.54)	0.12	2.25	(1.00, 3.03)	0.04
Unemployed	0.56	(0.12, 2.66)	0.47	0.59	(0.17,2.07)	0.41
Employed	ref			ref		
Ubudehe* categories						
I (poorest)	3.51	(0.73, 16.97)	0.12			
II (poor)	1.39	(0.72, 2.69)	0.33			
III &IV (wealthy)	ref					
**Insurance Type**						
Community Based Health Insurance or *Mutuelle*	4.29	(0.73, 16.97)	0.06	3.40	(1.21, 9.60)	0.02
Private	ref			ref		
**Parity**						
Primiparous (1)	ref					
Multiparous (2–5)	1.20	(0.59, 2.46)	0.61			
Grand-multiparous (> 5)	0.48	(0.15, 1.58)	0.23			
**Mode of transport (health center to hospital)**						
Ambulance	ref					
Walked	1.03	(0.50, 2.13)	0.93			
**Travel time, from home to health center**	1.00	(0.99, 1.00)	0.31			
**Household size**	0.79	(0.65, 0.96)	0.02	0.78	(0.66, 0.91)	< 0.01

^*^4-tier wealth system, the bottom tier represents the least privileged and the upper tier is for the financially better off

## Discussion

To our knowledge, this is the first study in a low-income country setting to estimate the comprehensive patient-level cost of c-section while including the cost of follow-up. We reported the direct medical cost of c-section of US$8.8, the cost of post-discharge follow-up of US$77.5 and the overall patient-level cost of c-section of US$122.2 during 30 days after-delivery. Over a third of women experienced financial catastrophe due to the costs associated with post-discharge care alone, and when combined with costs incurred prior to discharge, c-sections were financially catastrophic for over three-quarters of women. The vast majority of women who delivered at Kirehe District Hospital were poor prior to surgery and the cost of cesarean section was further impoverishing to 6.4% of women.

The direct cost of c-section found in this study is significantly lower compared to the costs of c-section in Mali (US$152.0), Nigeria (US$246.0), and the Democratic Republic of Congo (US$79.7) [10, 18, 32]. The substantially lower out-of-pocket payments for c-sections in Rwanda most likely reflects the cost cushion provided by Rwanda’s robust health insurance system. In our study, nearly all c-section patients were insured through CBHI. Although CBHI may offset the direct medial costs of c-sections, previously estimated in Rwanda as US$339.0 from the health facility perspective [11], c-section patients paid an additional US$31.4 in non-medical costs while still in the hospital. Moreover, the direct and indirect cost of c-section up to the time of discharge was catastrophic for 27.0% of women, which is comparable to a previous report of CHE among peritonitis patients in Rwanda [19]; but lower compared to the 60.0% incidence of CHE from c-section reported in India [33].

Surprisingly, the full cost of c- section rose to US$122.2 and was catastrophic for 77% of women when follow-up costs were considered. The tripling of out of pocket expenditures by follow-up costs implies that the full financial picture of c-section care can only be truly understood when post-discharge costs are examined. Most costs covered by health insurance globally are expenditures linked to direct medical care, but this misses the substantial follow-up costs. For example, despite full coverage of direct medical cost for people in *Ubudehe 1,* the incidence of CHE in this group within 30 days post-cesarean was 74.0%. Interestingly, women in *Ubudehe* 2 had the highest risk of CHE during their hospital stay, reflecting their increased vulnerability of their low incomes combined with lower coverage of expenses by CBHI. In fact, having membership in CBHI was associated with 2.40 times higher odds of CHE when follow up costs were accounted. This corroborates the argument that health insurance that covers direct medical costs, though essential, is not sufficient to financially protect poor patients [35, 36].

While the majority of women in this study were already poor, c-section delivery exacerbated financial hardship of poor women and threatens their living standards, as reflected by the fact that more than two-thirds of women sold assets or borrowed money to afford c-section surgery and hospitalization. The sales of property in order to afford obstetric surgery care in rural Rwanda was found to be higher than in Ethiopia (4.4%) [37].

Major contributors to overall c-section costs included post discharge lost wages (55.3%), costs of post discharge follow up at health center (15.8%), costs of caregiving (13.3%), and transportation costs (11%). Similarly, major cost drivers of post-discharge expenditure included lost wages (84.7%) and transportation (11.5%). Follow-up interventions and models that reduce lost wages and eliminate transportation costs may contribute to a reduction in CHE [16, 38, 39]. Examples of innovative holistic interventions including transportation interventions, like the Uganda Reproductive Health Voucher Project, [41], can be adapted to the local context. We are also exploring innovative mHealth strategies and contextualized community-based follow-up strategies that allow for home-based care to reduce the cost of follow-up as well as the physical burden of traveling [42].

Addressing lost wages will be challenging. While paid maternity leave for women employed in formal work sectors is a national policy in Rwanda [43], the majority of patients in rural Rwanda are farmers, do not have higher educational attainment and thus do not have access to these job-protected maternity leave packages. This may partly be explained by our finding of 2.25 times higher odds of CHE among farmers compared to those with formal employment; and 2.35 times higher odds of CHE among those who completed primary school compared to those with high school or more education. While efficient execution of Rwanda's community-based health insurance policies serves as a model for other LMICs, it does not cover the key drivers of the overall cost including lost wages and transportation, and thus does not offer full financial protection [44]. Creative health and social financing frameworks should explore options for maternity protection schemes for women working in the informal economy, through social insurance funds or cash transfer schemes [45]. C-sections have been found to impose further health costs if mothers return to work prior to recovery [46], and further studies are needed to explore the appropriate time to resume work after c-cesarean section, with consideration to mitigating lost wages in both the formal work sectors and for farmers.

These findings highlight a need for a more financially protective health financing framework for women who deliver surgically in rural settings. However, similar studies in other field of surgical specialties are necessary to provide a more holistic picture of the relationship between surgical follow-up costs and catastrophic expenditure in Rwanda. Studies in urban settings are also needed to paint a holistic picture of the cost of c-sections for women in both rural and urban settings. A broader view is necessary before specific and wide-reaching policy reforms can be suggested. Modelling studies on the implications of various health policy reforms can help identify viable financial protection frameworks that reduce follow up costs and lost wages. However, the direction of reform should consider covering transportation costs, minimizing of disruptions of caregivers' work, and reimbursement for lost wages as priorities.

Our findings should be interpreted in light of some limitations. Firstly, the calculations of household expenditure depended on patient memory and based on predetermined expense categories. Patients may have failed to accurately report expenditures or missed expenses that did not align with a category. Secondly, estimates of the cost of post-discharge follow-up were based on reports from patients who decided to seek care on their own, because currently, there is no uniform protocol for post-c-section follow-up. Thus, our findings do not fully reflect the true cost of c-section follow, if such follow-up protocols existed. We also recognize that our findings focus on a single district hospital in a single country; however, we believe this is generalizable to much of rural Rwanda and other parts of Africa because of similar post-c-section follow-up norms and similar economic and access challenges. Further, since our study only included individuals during a 5-month period, we may miss some seasonal patterns, for example changes in income due to agricultural cycles in this largely subsistence farming population or changes in costs such as transport which may vary with rainy seasons.

## Conclusion

When full costs are considered, c-section care confers significant risk of financial catastrophe on already impoverished households in rural Rwanda, despite the presence of a robust and widespread CBHI policy. Indirect non-medical cost and the full cost of follow-up for c-section from a patient perspective exceeds that of receiving initial medical care, and must be considered in development of policy and relevant interventions. Modelling of the financial implication of various follow up strategies should be encouraged to determine the most efficient and financially protective models of cost subsidy. Maternity protection schemes for women in informal work sectors should be explored to mitigate lost wages associated with extended period of recovery from surgical childbirth.

## Ethics statement

This study had ethical approval from the Rwandan National Ethics Committee (Kigali, Rwanda, No.326/RNEC/2019) and Harvard Medical School (IRB18-1033). Adult patients were read information about the study and voluntarily signed the consent prior to enrollment. We obtained voluntary assent from individuals less than 18 years, with signed consent from their parents or guardians.

## Data Availability

Data is available upon a reasonable request, by emailing niyianne@gmail.com.

## Abbreviations

C-section: Cesarean section
CHE: Catastrophic Health Expenditure
POD: Post-Operative Day
aOR: Adjusted Odds Ratio
IQR: Interquartile range
95% CI: 95% Confidence Interval
CBHI: Community-Based Health Insurance
REDCap: Research Electronic Data Capture
OpenMRS: Open Medical Record System
RWF: Rwandan Franc
LMICs: Low- and Middle-Income countries
